# The emerging role of platelet-rich plasma subepithelial infusion for vocal fold scar, sulcus, and atrophy

**DOI:** 10.1016/j.bjorl.2025.101716

**Published:** 2025-09-11

**Authors:** Gustavo Polacow Korn, Michal M. Johns

**Affiliations:** a Universidade Federal de São Paulo, São Paulo, SP, Brazil; b USC Voice Center, Caruso Department of Otolaryngology, Head and Neck Surgery, Los Angeles, United States of America

Great advances in the treatment of voice disorders have occurred over the past two decades particularly in the domain of minimally invasive laryngeal procedures. Despite this progress, challenges remain in the treatment of vocal fold Superficial Lamina Propria (SLP) deficiencies seen in vocal fold scar, sulcus and atrophy. A wide variety of interventions have been described all with significant limitations. Vocal fold corticosteroid subepithelial infusion, while safe, does not lead to great gains in restoration of pliable vocal fold mucosa.^1^ More invasive procedures, such as autologous tissue (fat/fascia) implantation and mucosal rearrangement/grafting procedures, carry risks of injuring remaining healthy vocal fold SLP and thus are often avoided outside of severe presentations of disease.

Tissue rejuvenation through the use of growth factors, scaffolds, and stem cells hold great promise for overcoming current limitations in the treatment of vocal fold scar, sulcus, and atrophy. More specifically, vocal fold subepithelial infusion using bFGF has been shown to improve vocal fold vibratory function in patients with vocal fold scar, sulcus and atrophy.^2^ Unfortunately, off the shelf growth factor therapeutics are not available in most regions of the world.

Platelet-Rich Plasma (PRP) is an autologous concentrate rich in platelets and growth factors, used successfully in regenerative medicine in orthopedics’, facial plastics, dermatology and hair restoration. PRP is obtained via peripheral blood collection and centrifugation, eliminating the risk of immunogenic reaction.^3^ It represent a low-cost and readily available source of autologous growth factors, cytokines, chemokines, and fibrinogen.^4^^,^^5^ PRP is injected into the SLP via subepithelial infusion in order to mechanically expand the vocal fold cover, disrupt scar adhesions, and deposit PRP for therapeutic effect. The procedure can be performed transorally, transcervically or transnasally via the working channel of a flexible endoscope. PRP injection appears to offer some antifibrotic effects and enhance vibratory function, possibly through their ability to combat fibrosis and promote regenerative healing.^3^

There are limited publications showing the short and long term benefits of the PRP injection in the vocal fold.^3^^,^^4^^,^^6^ Santa Maria et al.^4^ in a prospective trial with 15 patients with sulcus and scar that received 55 injections, without adverse effects, showed improvements in Patient Reported Outcome Measures (PROMs), quality of voice and mucosal wave evaluation. Woo and Murry,^6^ in a study with serial injection in 11 patients with recalcitrant, bilateral phono-traumatic lesion, showed a short-term (3 months) voice improvement.

However, current published evidence remains nascent and there is no standardized method of obtaining and processing PRP.^3^ And the durable effects remain unclear.^5^ These points raise concerns about the consistency and generalizability of the treatment. Regulatory councils and agencies may impose restrictions on the performance of this procedure until more evidence is obtained. Moreover, optimal patient selection criteria remain undefined. Woo, in a cohort of 63 patients with scar, atrophy or sulcus, observed that patients with severe dysphonia were less likely to experience clinical benefit compared to those with mild to moderate dysphonia.^5^

Well-designed prospective studies with inclusion of a control or placebo group are warranted to evaluate both the short- and long-term outcomes of PRP in the treatment of vocal fold scar, sulcus and atrophy. More standardization of PRP preparation techniques, and better characterization of PRP composition are essential to elucide the mechanism of action, optimize therapeutic protocols, and identify which patients are most likely to benefit from this promising regenerative approach for these challenging conditions.

## ORCID ID

Gustavo Polacow Korn: 0000-0003-3718-204X

Michal M. Johns III: 0000-0002-6273-569X

## Funding

None.

## Declaration of competing interest

The authors declare no conflicts of interest.
